# Accepted standards on how to give a Medical Research Presentation: a systematic review of expert opinion papers

**DOI:** 10.3205/zma001088

**Published:** 2017-02-15

**Authors:** Christine Blome, Hanno Sondermann, Matthias Augustin

**Affiliations:** 1 University Medical Center Hamburg-Eppendorf (UKE), Institute for Health Services Research in Dermatology and Nursing (IVDP), German Center for Health Services Research in Dermatology (CVderm), Hamburg, Germany

**Keywords:** scientific talks, scientific presentations, scientific conferences, lecturing, PowerPoint

## Abstract

**Background: **This systematic review aimed to extract recommendations from expert opinion articles on how to give a medical research presentation on a scientific conference and to determine whether the experts agree on what makes an effective or poor presentation.

**Methods: **Presentation-related terms were searched within article titles listed in PubMed, restricting the search to English-language articles published from January 1975 to July 2015. Recommendations were extracted from the articles, grouped by content, and analyzed for frequency. Ninety-one articles were included. Among 679 different recommendations, 29 were given in more than 20% of articles each. The five most frequent recommendations were to keep slides simple, adjust the talk to the audience, rehearse, not read the talk from slides or a manuscript, and make eye contact.

**Results: **No article gave advice that was the complete opposite of the 29 most frequent recommendations with the exception of whether a light or dark background should be used for slides.

**Conclusions:** Researchers should comply with these widely accepted standards to be perceived as effective presenters.

## Zusammenfassung

**Hintergrund:** Ziel dieser systematischen Übersichtsarbeit war es, aus publizierten Expertenstellungnahmen Empfehlungen zur Vorgehensweise bei medizinischen Präsentationen auf wissenschaftlichen Fachtagungen zu extrahieren und abzuleiten, ob Experten in der Frage übereinstimmen, was eine gute oder schlechte Präsentation ausmacht.

**Methoden: **Präsentationsbezogene Schlagwörter wurden in den Titeln englischsprachiger, in PubMed geführter und zwischen Januar 1975 und Juli 2015 erschienener Artikel gesucht. Aus den gefundenen Expertenartikeln wurden Empfehlungen extrahiert, inhaltlich gruppiert und nach Häufigkeit ausgewertet. Einundneunzig Artikel wurden eingeschlossen. Von insgesamt 679 unterschiedlichen Empfehlungen fanden sich 29 jeweils in mindestens 20% der Artikel. Die fünf häufigsten Empfehlungen lauteten: Einfache Folien verwenden; die Zuhörerschaft kennen; Augenkontakt halten; die Präsentation üben; nicht von Folien oder Manuskript ablesen.

**Ergebnisse:** In keinem Artikel wurde eine Empfehlung gegeben, die das klare Gegenteil einer der 29 häufigsten Empfehlungen darstellten, bis auf die Frage, ob ein heller oder dunkler Folienhintergrund verwendet werden sollte.

**Schlussfolgerung: **Wissenschaftler sollten sich an die hier gefundenen, weithin akzeptierten Empfehlungen halten, damit ihre Präsentationen positiv wahrgenommen werden.

## 1. Introduction

Some presentations at medical conferences are easy to follow, engaging, and even inspire changes in the way patients are treated or scientific work is conducted. Conversely, others induce the audience to check their mobile phones or take a nap because they are so difficult to concentrate on. 

What exactly makes great medical research presentations great? Empirical or even experimental data on this question are scarce [1], [2], [3], [4]. However, more than 80 authors of expert opinion articles have described what they believe a medical presenter should or should not do. The aim of this review was to extract all recommendations from these articles and determine whether the experts agree on what makes a medical research presentation either effective or poor. 

## 2. Methods

Parts of this study were obtained from a previous dissertation by Sondermann, 2014 [5].

Presentation-related terms were searched within the titles of articles listed in PubMed, restricting the search to English-language articles published from January 1975 to July 2015. The search terms were:

(scientific[ti] AND presentation*[ti]) OR (conference[ti] AND presentation*[ti]) OR (oral[ti] AND presentation*[ti]) OR (research[ti] AND presentation*[ti]) OR (scientific[ti] AND meeting*[ti]) OR (public[ti] AND speaking[ti]) OR (public[ti] AND speech[ti]) OR (Power[ti] AND Point[ti]) OR PowerPoint[ti] OR (scientific[ti] AND talk*[ti]) OR lecturing[ti] OR lectures[ti] OR (scientific[ti] AND conference*[ti]) OR (medical[ti] AND presentation*[ti]) OR (paper[ti] AND presentation*[ti]) AND "1975/01/01"[PDAT]:"2015/07/31"[PDAT] AND English[lang]

The bibliographies of eligible articles were reviewed for further references. 

We included expert opinion articles and editorials that provided advice on how to give a medical research presentation at scientific conferences. We excluded articles exclusively referring to lectures to students, continued medical education, or health care management.

Recommendations were extracted from each article, including both direct (e.g., “You should…”) and indirect recommendations (e.g., “Remember the audience’s time (…) should not be abused by presentation of uninteresting preliminary material” [6]). Mere suggestions were not extracted; these were typically signaled by words such as “consider.” We also excluded recommendations on abstract writing, use of outdated technology (e.g., diapositives), radiologic images (for being too specific), and technical aspects (e.g., choice of software). 

Differently worded advice from two authors was regarded as the same recommendation if equal in content (e.g., “initially, rehearse alone” [7] and “initially, practice the talk alone” [8]). Similar recommendations were grouped into more general but still concrete advice. For example, “limit the number of lines on a slide to six” [9] and “no more than seven lines per slide” [10] were grouped into “limit the number of lines per slide.” Finally, we determined the frequency of recommendations, counting those given in two articles by the same first author only once.

## 3. Results

The PubMed search delivered 4,140 hits, 91 of which met the inclusion criteria [6], [7], [8], [9], [10], [11], [12], [13], [14], [15], [16], [17], [18], [19], [20], [21], [22], [23], [24], [25], [26], [27], [28], [29], [30], [31], [32], [33], [34], [35], [36], [37], [38], [39], [40], [41], [42], [43], [44], [45], [46], [47], [48], [49], [50], [51], [52], [53], [54], [55], [56], [57], [58], [59], [60], [61], [62], [63], [64], [65], [66], [67], [68], [69], [70], [71], [72], [73], [74], [75], [76], [77], [78], [79], [80], [81], [82], [83], [84], [85], [86], [87], [88], [89], [90], [91], [92], [93], [94], [95], [96]. Of the 91 articles, 63 were from the medical field and 28 from related fields such as nursing. We found 3 to 103 different recommendations in each article, totaling 3,135 recommendations. Identification of identical recommendations and grouping similar ones resulted in 679 different recommendations. Of these, 349 were given in only one article each; for example, “remain in the hall from the start of the session until your talk” [94].

The most frequent advice, given in 62.9% of articles, was to keep slides simple. In particular, authors stated that one should not overload slides or include too much detail, but use clear, concise, simply designed visuals instead. Simplicity of visuals was also the subject of 5 of the 29 most frequent recommendations (see Table 1 (Tab. 1)), including limiting the number of lines per slide (42.9%) and number of words per line (28.6%), using simple tables and graphs (34.1%), using animations carefully (27.5%), and putting phrases, not sentences, on slides (24.2%).

The second most frequent advice, to know one’s audience (52.7%), referred to who the audience is (e.g., profession, size, age, education), what they already know of the topic, or why they are there (i.e., what their expectations, attitudes, and interests are). Authors advised adjusting the presentation accordingly instead of using canned talks.

Making eye contact was the third most frequent advice (46.2%). This was specified by some authors as making eye contact with many or all persons, making eye contact with persons in all sections of the audience, or making continuous eye contact.

Rehearsal of the presentation was recommended in 44.0% of the articles. In addition, one-third of the articles advised rehearsal in front of other persons. Taken together, 56.0% of the articles gave at least one of these two recommendations. Timing the presentation beforehand – recommended by 38.5% – can ensure that the presenter will stick to the allotted time, an advice given by 40.7%. Further advice calling for thorough preparation was to know one’s topic “like the back of one’s hand” (31.9%), to develop an objective for the talk (28.6%), to and prepare for questions (20.9%). All equipment should be tested beforehand (27.5%).

When delivering the presentation, one should not read the talk from either slides or a manuscript (44.0%). For this purpose (and for simplicity) slides should contain words or phrases instead of complete sentences (24.2%). 

The presenter should vary the presentation of his or her voice instead of speaking monotonously (29.7%), not speak too fast (24.2%), face the audience (23.1%), and show some enthusiasm, excitement, or energy (20.9%). To enhance understanding, one should keep the presentation clear and simple (26.4%), be logical (23.1%), and end with a summary (26.4%). The number of slides should be limited (27.5%); most articles specified one slide per minute (n=7, 7.7%).

The slides should be readable (42.9%), referring to both text and visuals. This was probably also the reason for recommending large font sizes (this advice was not included in the 29 most frequent recommendations, however; n=18, 19.8%). Authors generally disagreed regarding the exact size to be used, which ranged from 18 to 32 points; a font size of 24 points was recommended most frequently (n=8, 8.8%).

Authors agreed that the slide design should be consistent throughout the presentation (20.9%) and that contrasting colors should be used (20.9%). Most authors recommended using a dark background (26.4%), while only few recommended using a light background (n=3, 3.3%), arguing that this makes slides easier to read [15], [46]; one paper [89] recommended light background for charts and graphs, but not for text slides (without giving reasons).

None of the included articles gave advice that was the complete opposite of these 29 most frequent recommendations (except for the light versus dark background). However, limiting advice was occasionally given, such as not to practice too much in order to save some enthusiasm [62] or not to exceed >10% of the original time [19]. Authors also disagreed on a few topics that did not make it to the 29 most frequent recommendations, including whether clipart or cartoons should be included, whether using a pointer is recommended, and whether information should be added sequentially on a slide.

## 4. Conclusions

This review extracted recommendations from 91 expert opinion articles on how to give a medical research presentation. We found a high degree of concordance among authors, with 29 recommendations given in more than one-fifth of articles each and very little explicit discordance.

Our findings are limited by the fact that we restricted the literature search to one database and to article titles (without the latter, our search would have yielded 195,766 hits). Nevertheless, we included 91 articles on the presentation of medical research and thus considerably more than two previous reviews, which included 9 expert opinion articles on podium presentations each [97], [98]. 

In addition, the distinction between what authors meant to be recommendations versus mere suggestions was a matter of interpretation; the same is true for decisions on whether recommendations were similar enough to be grouped.

The fact that many authors recommend a behavior does not necessarily mean it will indeed be effective. This can be tested in experimental studies that systematically vary a presenter’s behavior. As in clinical studies, the outcome of interest would need to be defined, which is rarely done in expert opinion articles. We propose as “presenter-relevant outcomes” a) to induce learning effects (i.e., comprehension and retention [99]), b) to change attitudes, c) to interest and entertain, and d) to improve the presenter’s reputation (e.g., by appearing competent). 

To our knowledge, experimental studies have only been done for presentations other than medical research presentations. Surprisingly, the recommendation given most often in this study, “keep your slides simple”, has not been supported with regard to the amount of text on a slide (an aspect also related to further recommendations, like “limit the number of lines per slide”, “limit the number of words per line”, and “put phrases, not sentences, on slides”). A number of studies in students did not find significant differences in retention of information after presentations with concise slides as compared to presentations with more detailed slides [100], [101], [102], as would have been expected by cognitive load theory. This theory states that information will not be encoded adequately if the capacity of our working memory is overloaded [103], [104], for example when trying to understand detailed slides and at the same time listen to the presenter. These surprising findings underline the necessity of experimental research on presentation techniques. However, simple slides have been found to be more effective with regard to a different aspect: that is, whether they include pictures not related to the content of the talk. Here, recall was better in students who attended a presentation using slides with irrelevant pictures [105].

The third most frequent advice, to make eye contact, was found to be effective in one study: Not only did students consider a speaker who made eye contact to be more credible and his talk to be more comprehensible, but they actually learned more as indicated by a subsequent multiple-choice test [102]. In this study, the “eye contact” condition also differed from the control condition in that the presentation was more lively (recommendation no. 13: “vary your voice“) and in that the presenter did not read from written text only but also made colloquial interjections (recommendation no. 5: “do not read the talk from slides or a manuscript”). 

It is quite possible that empirical studies will contradict the advice found in this opinion-based study. For example, there is reason to assume that dark backgrounds (recommended by 24 experts as compared to 3 experts recommending light background) may have disadvantages. For example, they may require dimming the lights so that the audience can read the slides, which in turn may lead to reduced levels of attention due to increased tiredness.

In addition, findings from previous studies may not be generalizable to medical conference presentations where the audience may differ in important aspects from students (which have been the subjects of many of the experiments [106]) – for example with regard to their reasons for attendance and their prior knowledge of the topic. Future experimental studies should therefore investigate whether the recommendations found in this study are indeed effective, looking at different audiences and contexts, and focusing also on rarely explored aspects related to the preparation of the presentation, like adjustment of the talk to the specific audience (recommendation no. 2) and rehearsal (recommendation no. 4).

Probably one of the main reasons that a particular piece of advice was given in the expert opinion papers is that the authors believed that many presenters did not yet follow it. The 29 most frequent recommendations can thus be interpreted as the 29 most common mistakes made by conference presenters. Most of them appear to be common sense and are generally well known [99]; therefore, why are flaws so common, even in senior presenters [98]? Researchers may be unwilling to invest time in thorough preparation [107], or perhaps they have competing interests such as drawing the audience’s attention away from themselves or using slides as a memory aid [104]. However, if presenters want their talk to be inspiring and practice-changing, they should adhere to the agreed advice found in this review.

Future experimental studies should investigate the effectiveness of the recommendations found in this opinion-based review.

## Funding sources

The authors have no funding sources to declare.

## Authors' contributions

CB conceived of the study, participated in its design, conduction, and analysis, and drafted the manuscript. HS participated in the study design, conduction, and analysis and helped draft the manuscript. MA participated in the study design. All authors read and approved the final manuscript.

## Competing interests

The authors declare that they have no competing interests.

## Figures and Tables

**Table 1:**
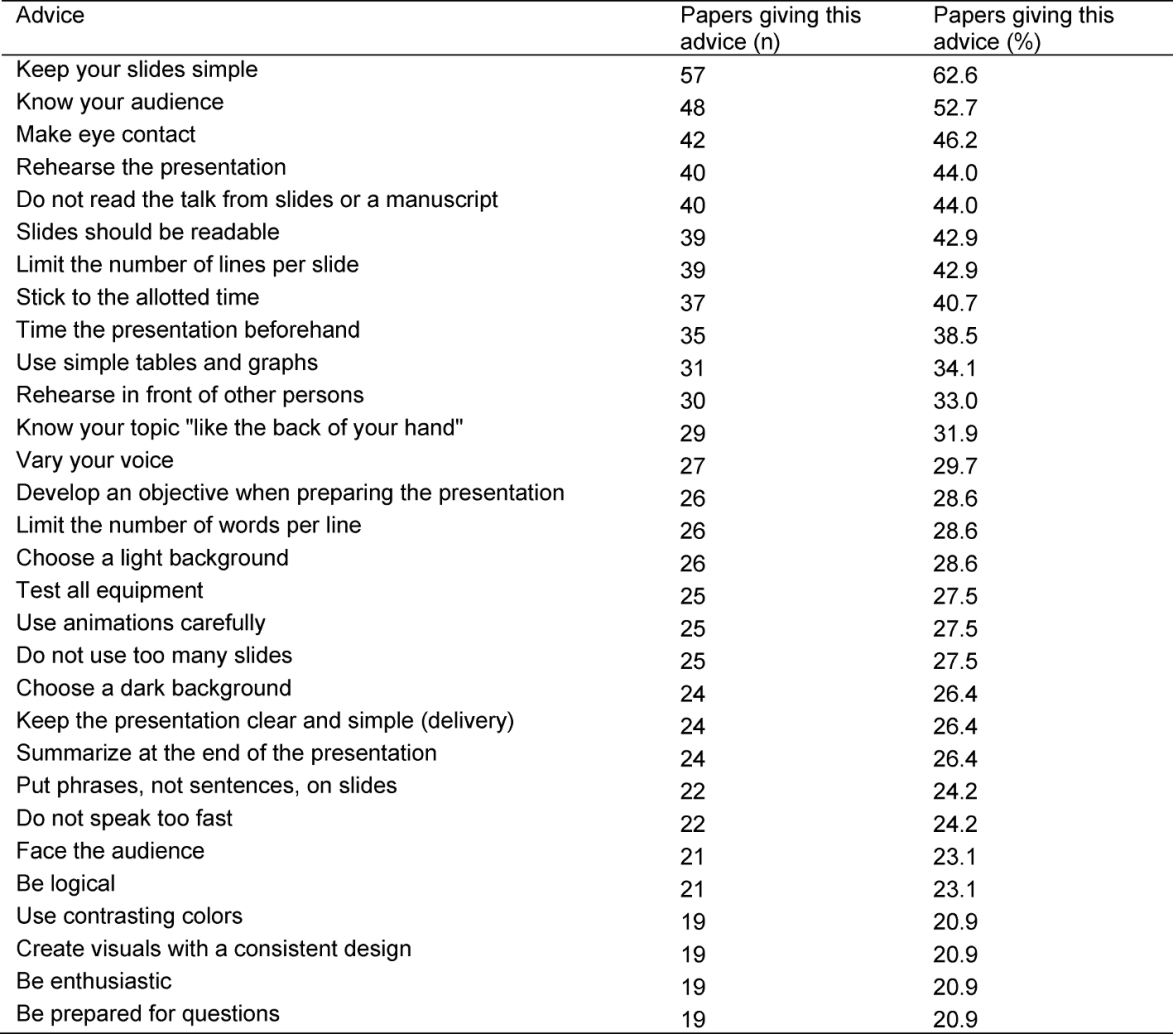
Most frequent recommendations on how to give a medical research presentation (29 recommendations given in ≥ 20% of included articles each)
